# Owner perceived differences between mixed-breed and purebred dogs

**DOI:** 10.1371/journal.pone.0172720

**Published:** 2017-02-21

**Authors:** Borbála Turcsán, Ádám Miklósi, Enikő Kubinyi

**Affiliations:** 1 Department of Ethology, Eötvös Loránd University, Budapest, Hungary; 2 Institute of Cognitive Neuroscience and Psychology, Research Centre for Natural Sciences, Hungarian Academy of Sciences, Budapest, Hungary; 3 MTA-ELTE Comparative Ethological Research Group, Budapest, Hungary; University of Lethbridge, CANADA

## Abstract

Studies about the behaviours of mixed-breed dogs are rare, although mixed-breeds represent the majority of the world’s dog population. We have conducted two surveys to investigate the behavioural, demographic, and dog keeping differences between purebred and mixed-breed companion dogs. Questionnaire data were collected on a large sample of dogs living in Germany (N = 7,700 purebred dogs representing more than 200 breeds, and N = 7,691 mixed-breeds). We found that according to their owners, mixed-breeds were (1) less calm, (2) less sociable toward other dogs, and (3) showed more problematic behaviour than purebreds (p < 0.001 for all). Mixed-breeds and purebreds were similar in trainability and boldness scores. However, twelve out of 20 demographic and dog keeping factors differed between purebred and mixed-breed dogs, and two factors showed considerable (> 10%) differences: neutering was more frequent among mixed-breeds, and they were acquired at older ages than purebreds (p < 0.001 for both), which could result in the observed behaviour differences. After controlling for the distribution of the demographic and dog keeping factors, we found that mixed-breeds were (1) more trainable than purebreds, (2) less calm, and (3) showed more problematic behaviour than purebreds (p < 0.001 for all). We discuss that these differences at least partly might be due to selective forces. Our results suggest that instead of being the “average” dogs, mixed-breeds represent a special group with characteristic behavioural traits.

## Introduction

When it comes to selecting a new canine companion, choosing an incompatible breed could have dire consequences, regarding the well-being of both the owner and the dog. Although the typical behaviour of different dog breeds has attracted considerable scientific attention (e.g. [1]), studies about mixed-breed dogs are rare. Mixed-breed dogs comprise dogs of heterogeneous origin that by definition, belong to no recognized breed, and their ancestry is usually complex or unknown. They could be intentionally bred by humans as hybrids of recognized breeds (e.g. ‘designer dogs’), they could be offspring of a purebred and a mixed-breed dog, offspring of two mixed-breeds, or descendants of stray, feral or pariah dog populations.

The percentage of mixed-breed dogs (out of all dogs living in households in the USA), is estimated to be around 53% by the American Veterinary Medical Association [2], and 44% by the American Pet Products Association [3]. In Germany and in the UK, approximately 31–33% of dogs are mixed-breeds [4,5], while in Australia, mixed-breeds make up half of the population of dogs living in human households [6]. In scientific databases, mixed-breeds compose approximately one third of the dogs [7–9]. However, these proportions are likely to underestimate the real number of mixed-breeds in the whole dog population, considering that these data estimate only the “owned” dog population (based on pet industry reports, veterinary records, household panels or mail surveys, [10]). The mixed-breeds’ contribution to the stray, feral and pariah dog populations is hard to estimate reliably. However, they probably represent the majority of dogs worldwide [11].

Mixed-breeds are often assumed to have some phenotypic advantage over purebreds in terms of fitness (e.g. improved health and lower susceptibility to diseases), because they show a lower level of homozygosity and have much higher genetic variation [12–14], which could lead to hybrid vigour [15]. Some studies have reported that adult mixed-breed dogs are less likely to suffer from inherited genetic diseases and live longer than purebreds [12,16–18]. Several studies have detected behavioural differences between mixed-breeds and purebred dogs. For example, Bennett and Rholf [7] reported mixed-breeds to be more disobedient, more nervous, more excitable, and exhibited excessive barking more frequently in the case of mixed-breeds than in purebred dogs. Hsu and Sun [19] reported higher ranks for three aggression subscales in mixed breeds (towards strangers, towards dogs, and towards owner/s). Mixed-breeds have also been reported to have an increased risk to develop noise phobia [20], they were more likely to be aggressive toward unfamiliar people, more fearful, and more sensitive to touch than purebreds [21]. Temesi et al. [22] found higher neuroticism, dog-directed fear and human-directed fear in mixed-breeds than in all AKC breed groups except the Toy dogs group. On the other hand, Ottenheimer-Carrier et al. [23] did not find any differences between purebred and mixed-breed dogs in three personality assessments.

One should note, however, that the main aim of these studies was not to compare purebreds and mixed-breeds. Therefore, these results could reflect a number of other systematic differences between these dog groups apart from the dogs’ purebred status. For example, dog keeping practices have been reported in association with numerous behaviour traits (e.g. [9,24–26]), therefore differences in these factors could result in behaviour differences between mixed-breeds and purebreds.

In the current study, we explored possible differences between mixed-breeds and purebreds in various behaviour traits and dog keeping characteristics. We hypothesized that when numerous individuals from many breeds are investigated together, breed-specific behavioural characteristics may balance each other out. Therefore, after controlling for differences in dog keeping practices between mixed-breeds and purebreds, we expected no differences between the mean behavioural trait scores of a large population of mixed-breed dogs and purebred dogs. The gene flow between the two populations also favours this hypothesis. Purebreds generally originated from ancient mixed-breeds and mixed-breeds often have purebreds among their ancestors.

Two surveys were developed, both measuring the demographic characteristics of the owners and dogs, as well as the dog keeping practices. Survey 1 aimed at measuring the dogs’ general behaviour tendencies (personality), and Survey 2, typical behaviour problems.

## Materials and methods

### Ethics statement

We collected the data using an online questionnaire designed to assess the dogs’ behaviour via owner report. According to the currently operating Hungarian law (‘‘1998. évi XXVIII. Törvény”—the Animal Protection Act, 3^rd^ paragraph, 9^th^ point), non-invasive observational experiments on dog behaviour are not considered as animal experiments, and are therefore allowed to be conducted without any special permission from the University Institutional Animal Care and Use Committee (UIACUC). The filling out of the questionnaires was voluntary and anonymous so the study did not violate respondents’ privacy. Informed consent was included in the introductory letter of the questionnaires.

### Subjects

We used the questionnaire method because it allowed us to collect data from a large number of subjects, which were highly diverse in terms of breed and dog keeping practices. A total of 14,004 dog owners filled out the first survey, and 10,240 filled out the second. We excluded reports with missing data and repetitions (where owners filled in two or more reports about the same dog, we used these data only for calculating test-retest and inter-rater reliability). There were N = 312 owners who filled in both surveys, their demographic and dog keeping questions were considered only once. We grouped the dogs into purebred and mixed-breed groups based on the owners’ specification. To control for the effect of breed popularity in the purebred group we defined a cut-off point for both surveys, so that the maximum number of individuals in a given breed was N = 60 for Survey 1, and N = 37 for Survey 2. We determined the cut-off point to match the total number of individuals in the purebred and mixed-breed group. If a breed was represented with more individuals than the cut-off point, we selected a random sample for the final dataset.

The final sample of Survey 1 consisted of N = 9186 dogs (4593 in the purebred and 4593 in the mixed-breed groups), the purebred group was composed of 254 breeds and no breeds had more than 60 representatives. The final sample of Survey 2 had N = 6384 dogs (N = 3199 dogs in the purebred and N = 3185 dogs in the mixed-breed group), the purebred group was composed of 251 breeds and no breeds had more than 37 representatives. Descriptive information of the databases can be found in the supplemental material (S1 and S2 Tables).

### Procedure

We conducted two surveys in Germany, both of which were developed by Jesko Wilke, a freelancer journalist of the German ‘Dogs’ magazine. The data were collected online by the magazine’s own website (www.dogs-magazin.de). The results of Survey 1 have already been published in [9] and [27].

Both surveys comprised two parts. The first part collected information about the demographic characteristics of the owners and dogs, as well as about dog keeping practices. Twelve of these questions were the same in both surveys; eight were present in only one. The second part differed in the two surveys. Survey 1 aimed at measuring the dogs’ general behaviour tendencies (personality) and was developed based on a human personality inventory. This questionnaire contained 24 items (e.g. “My dog is calm, even in ambiguous situations”), and for each item the owners were asked to indicate their level of agreement on a 3-point scale (true, partly true, and not true) (see S3 Table). Our previous results using principal component analysis have revealed that 17 items out of the 24 belonged to four components, labelled as calmness, trainability, dog sociability, and boldness, all traits with middle or high internal consistency ([9,27], S3 Table).

Survey 2 listed 12 examples of typical behavioural problems such as “My dog usually does not listen to me when I call him/her back” (S4 Table). Again, the owners indicated for each statement how much they agree with it using a 3-point scale. The questions were designed to assess not only the prevalence of behavioural problems of the dogs, but also the owners’ attitude towards these behaviours; i.e. if he/she considers them as problematic. The internal consistency of the 12 items of the “Problematic behaviour” scale (Cronbach’s alpha = 0.720) indicates that they form one single trait referring to how problematic the owners assess their dogs’ behaviour in general. We calculated the trait scores of Survey 1 and Survey 2 by taking the mean of the variables belonging to a given trait.

We used the multiple reports from the same owner about the same dog (N = 208 in Survey 1 and N = 280 in Survey 2), for calculating test-retest reliability, and reports collected from a second owner (of the same dog) (N = 85 in Survey 1 and N = 136 in Survey 2), for assessing inter-rater reliability of the surveys.

### Statistical analyses

#### Reliability analyses of the surveys

We analysed the test-retest and inter-rater reliability of the surveys using Intraclass correlations (test-retest: Two-Way Mixed model, consistency; inter-rater: One-Way Random model, absolute agreement).

#### Behaviour trait differences between the dog groups

The data were analysed at the individual level with each dog as a separate data point. To analyse the difference between the purebred and mixed-breed dogs, we compared the five behavioural traits (the four personality traits from Survey 1 and the Problematic behaviour trait from Survey 2), between the dog groups using independent sample t-tests, and the effect size was estimated using Cohen’s d.

#### Demographic and dog keeping differences between the dog groups

For the twelve questions which were common between the two surveys we pooled the data of the two surveys. The categorical variables were compared between the dog groups (purebred and mixed-breed) using Chi-squared tests with z post hoc test, and the age of the dogs in the two groups was compared using Mann-Whitney U test. For the comparisons of the demographic and dog keeping factors, we provided unstandardized effect size statistics (i.e. the magnitude of the difference between the groups); since we deemed them more meaningful in this case than standardized measures [28].

#### Relationship between the behaviour differences and demographic/dog keeping differences

We analysed the associations between the behavioural traits and demographic and dog keeping factors using five general linear models (GLM). In each model the dependent variable was the behavioural trait, and the explanatory factors included the dog group (purebred and mixed-breed) as a fixed factor, and all the demographic and dog keeping factors in which significant differences were found between the dog groups (age as a covariate, and categorical variables as fixed factors). The aim of these GLM analyses was not only to investigate how the demographic and dog keeping factors are associated with the behavioural traits, but also to investigate if the behaviour differences between the dog groups remain significant when controlling for any differences in the demographic and dog keeping factors. We also added all two-way interactions between the dog group and the demographic/dog keeping factors. A significant interaction would mean that a given factor has a different relationship with the behavioural trait in purebreds, than in mixed-breeds. Non-significant interactions were removed from the model sequentially in the order of their decreasing significance; however, all main effects, even non-significant ones, were left in the model. The effect size of each factor in the final model was estimated with partial eta squared, which reflects the proportion of total variation attributable to a given explanatory factor, when excluding other factors in the model from the total non-error variation [29].

In order to take into account the large number of subjects investigated, and the multiple statistical analyses performed, we set the threshold of the significance level to p = 0.00037 (0.05 /134) according to the Bonferroni correction method. Statistical analyses were performed with SPSS version 22.

## Results

### Reliability analyses of the surveys

Both the inter-observer reliability and the test-retest reliability of the five traits were excellent (Table 1).

**Table 1:** Reliability measures of the five behaviour traits.

		Calmness	Trainability	Dog sociability	Boldness	Problematic behaviour
Inter-observer reliability (ICC 1,k)						
	ICC	0.830	0.753	0.849	0.874	0.766
	F	F_84,85_ = 5.881	F_84,85_ = 4.046	F_84,85_ = 6.640	F_84,85_ = 7.939	F_135,136_ = 4.265
	p	< 0.001	< 0.001	< 0.001	< 0.001	< 0.001
Test-retest reliability (ICC 3,k)						
	ICC	0.899	0.886	0.944	0.912	0.911
	F	F_207,207_ = 9.923	F_207,207_ = 8.791	F_207,207_ = 17.788	F_207,207_ = 11.399	F_279,279_ = 11.206
	p	< 0.001	< 0.001	< 0.001	< 0.001	< 0.001

### Behaviour trait differences between the dog groups

In Survey 1, mixed-breed dogs were rated to be less calm (t-test, N = 9,186 t = 14.910; p < 0.001, Cohen’s d = 0.311), and less sociable toward other dogs (t = 4.919; p < 0.001, Cohen’s d = 0.103), than purebred dogs. We found no significant difference in trainability (t = 1.946; p = 0.052), or boldness (t = 0.519; p = 0.604) traits between the dog groups. In Survey 2, owners of mixed-breeds reported their dogs’ behaviour as more problematic (t-test, N = 6,384 t = 5.577; p < 0.001, Cohen’s d = 0.140), than the owners of purebreds.

### Demographic and dog keeping differences between the dog groups

We found significant differences between purebred and mixed-breed dogs in 12 out of the 20 investigated demographic and dog keeping factors (Table 2), after correcting for multiple comparisons. Ten of these factors were common between the two surveys, two factors were investigated in Survey 2 only. However, due to the large sample size, even with the p < 0.00037 threshold, a significant result in most of the factors indicated only a small (3–6%) difference in a given category between the dog groups. Regarding the demographic factors: mixed breed dogs were found to be older in our sample, and there were more females among them than among purebreds. Mixed-breeds’ owners were more likely to be women, they were younger, had lower level of education and had less previous experience with dogs than the owners of purebreds. However, we found no difference between the groups in the number of adults and children in the household.

**Table 2:** Comparison of demographic and dog keeping factors between purebred and mixed-breed dogs.

Factors in both surveys	Categories	Purebred N = 7,698	Mixed-breed N = 7,691	Statistics	Absolute magnitude of the difference
Dogs’ age in years (mean ±SD)	–	3.42 (±3.13)	3.68 (±3.24)	z = 5.83	0.26 years
				*p < 0*.*001*	
Dogs’ sex	Male	**57.2%**	52.7%	χ^2^ = 31.25	male: 4.5%
	Female	42.8%	**47.3%**	*p < 0*.*001*	female 4.5%
Dogs’ neuter status	Intact	**69.7%**	51.3%	χ^2^ = 544.84	intact: 18.4%
	Neutered	30.3%	**48.7%**	*p < 0*.*001*	neutered: 18.4%
Dogs’ age at acquisition	bred by owner	2.0%	1.6%	χ^2^ = 547.97	bred by owner: 0.4%
	2–12 weeks	**62.5%**	44.6%	*p < 0*.*001*	2–12 weeks: 17.9%
	3–12 months	20.7%	**28.3%**		3–12 months: 7.6%
	> 1 year	14.8%	**25.5%**		> 1 year: 10.7%
Dogs’ training experience	no training	32.7%	**41.6%**	χ^2^ = 168.24	no training: 8.9%
	1 type	25.7%	25.8%	*p < 0*.*001*	1 type: 0.1%
	2 types	**22.3%**	18.2%		2 types: 4.1%
	3 or more types	**19.3%**	14.4%		3 or more: 4.9%
Owners’ gender	Man	**20.0%**	16.4%	χ^2^ = 32.08	man: 3.6%
	Woman	80.0%	**83.6%**	*p < 0*.*001*	woman: 3.6%
Owners’ age	≤ 18 years	5.0%	4.8%	χ^2^ = 69.54	≤ 18 years: 0.2%
	19–30 years	25.9%	**31.2%**	*p < 0*.*001*	19–30 years: 5.3%
	31–60 years	**65.6%**	61.8%		31–60 years: 3.8%
	> 60 years	**3.5%**	2.2%		> 60 years: 1.3%
Owners’ education	primary school	20.6%	**22.0%**	χ^2^ = 20.63	primary: 1.4%
	secondary school	40.5%	40.8%	*p < 0*.*001*	secondary: 0.3%
	College	25.7%	26.4%		college: 0.7%
	University	**13.1%**	10.8%		university: 2.3%
N of previous dogs	no previous dog	39.7%	**46.2%**	χ^2^ = 178.30	no previous dog: 6.5%
	1 dog	25.5%	**28.0%**	*p < 0*.*001*	1 dog: 2.5%
	2 dogs	**16.0%**	14.0%		2 dogs: 2.0%
	3 or more dogs	**18.8%**	11.8%		3 or more dogs 7.0%
Purpose of keeping the dog	family member only	45.4%	47.1%	χ^2^ = 6.51	fam. mem. only: 1.7%
	family member + other	47.3%	46.3%	p = 0.039	fam. mem.+other: 1.0%
	not family member	7.3%	6.6%		not fam. mem.: 0.7%
N of people in the household	only 1 person	10.6%	11.8%	χ^2^ = 9.46	only 1 person: 1.2%
	2 people	45.1%	43.0%	p = 0.009	2 people: 2.1%
	3 or more people	44.3%	45.2%		3 or more: 0.9%
N of dogs in the household	no other dog	66.3%	**72.6%**	χ^2^ = 99.30	no other dog: 6.3%
	1 other dog	**20.7%**	18.8%	*p < 0*.*001*	1 other dog: 1.9%
	≥ 2 other dogs	**13.0%**	8.6%		≥ 2 other dogs: 4.4%
**Factors only in Survey 1**	**Categories**	**Purebred N = 4,593**	**Mixed-breed N = 4,593**	**Statistics**	**Absolute magnitude of the difference**
Hours spent with the dog / day	≤ 3 hours	27.0%	28.7%	χ^2^ = 3.41	≤ 3 hours: 1.7%
	> 3 hours	73.0%	71.3%	p = 0.065	> 3 hours: 1.7%
Frequency of playing / week	1–5 days	20.0%	21.8%	χ^2^ = 4.56	1–5 days: 1.8%
	6–7 days	80.0%	78.2%	p = 0.033	6–7 days: 1.8%
**Factors only in Survey 2**	**Categories**	**Purebred N = 3,199**	**Mixed-breed N = 3,185**	**Statistics**	**Absolute magnitude of the difference**
N of children in the household	1 or more	20.7%	21.4%	χ^2^ = 0.54	1 or more: 0.7%
	None	79.3%	78.6%	p = 0.462	none: 0.7%
Where the dog is kept	only indoors	71.3%	**76.7%**	χ^2^ = 24.25	only indoors: 5.4%
	in- and outdoors	**26.4%**	21.3%	*p < 0*.*001*	in- and outdoors: 5.1%
	only outdoors	**2.3%**	2.0%		only outdoors: 0.3%
Hours spend walking the dog	< 1 hour	**12.1%**	8.8%	χ^2^ = 25.17	< 1 hour: 3.3%
	1–3 hours	78.0%	78.8%	*p < 0*.*001*	1–3 hours: 0.8%
	> 3 hours	9.9%	**12.4%**		> 3 hours: 2.5%
Hours spend playing / day	≤ 1 hour	68.6%	68.7%	χ^2^ = 0.001	≤ 1 hour: 0.1%
	> 1 hour	31.4%	31.3%	p = 0.987	> 1 hour: 0.1%
Buy gifts for the dog	Yes	68.5%	65.8%	χ^2^ = 5.21	yes: 2.7%
	No	31.5%	34.2%	p = 0.023	no: 2.7%
Allow the dog into the bed	Yes	39.3%	37.6%	χ^2^ = 1.98	yes: 1.7%
	No	60.7%	62.4%	p = 0.160	no: 1.7%

For each categorical variable the proportion of the dogs falling into each category are presented separately for each dog group. Where significant group differences were found (as indicated by the Chi-squared tests with z post-hoc tests), the category with the larger proportion in a given dog group was marked in bold. Only differences with p < 0.00037 were considered significant (marked in italics). The absolute magnitude of the difference is presented as a measure of effect size.

Regarding the dog keeping factors: mixed-breeds were more likely to be neutered, owners acquired them at an older age, they received less training, and they were more likely to be kept only indoors and as single dogs, than purebred dogs. However, mixed-breeds’ owners walked their dogs for longer than the owners of purebreds. We found no difference between the groups in how much time the owners spend with their dog in general, or with playing, for what purpose the owners keep the dog, whether they buy gifts for the dog and whether the dog is allowed onto the bed. (Table 2).

### Relationship between the behavioural traits and the demographic/dog keeping factors

When controlling for all demographic and dog keeping factors where the difference between the dog groups was significant in our previous analysis (10 factors for the traits from Survey 1 and 12 factors for the Problematic behaviour trait from Survey 2, see Table 2), we found that the dogs’ breeding status was still significantly associated with the calmness and problematic behaviour traits (Table 3), and interestingly, it was also significant for trainability. However, the dog group did not remain as a significant main effect in the GLM models of dog sociability and boldness after the Bonferroni correction (p = 0.009 and p = 0.003, respectively).

**Table 3:** Relationship between the demographic and dog keeping factors and the behaviour traits investigated with GLMs.

		Calmness			Trainability			Dog sociability			Boldness			Problematic behaviour		
Source	df	F	p	partial eta^2^	F	p	partial eta^2^	F	p	partial eta^2^	F	p	partial eta^2^	F	p	partial eta^2^
Corrected Model		21.4	0.000	0.049	47.6	0.000	0.103	37.7	0.000	0.083	17.7	0.000	0.041	44.6	0.000	0.154
Dog group	1	*111*.*0*	*0*.*000*	*0*.*012*	*12*.*9*	*0*.*000*	*0*.*001*	6.9	0.009	0.001	9.1	0.003	0.001	*23*.*8*	*0*.*000*	*0*.*004*
Dogs’ age	1	*35*.*9*	*0*.*000*	*0*.*004*	*207*.*1*	*0*.*000*	*0*.*022*	*524*.*5*	*0*.*000*	*0*.*054*	5.0	0.025	0.001	*160*.*4*	*0*.*000*	*0*.*025*
Dogs’ sex	1	1.4	0.242	0.000	0.6	0.438	0.000	*26*.*7*	*0*.*000*	*0*.*003*	*128*.*1*	*0*.*000*	*0*.*014*	*28*.*4*	*0*.*000*	*0*.*004*
Dogs’ neutered status	1	*54*.*9*	*0*.*000*	*0*.*006*	6.8	0.009	0.001	1.2	0.278	0.000	5.7	0.017	0.001	0.2	0.630	0.000
Age at acquisition	3	*25*.*9*	*0*.*000*	*0*.*008*	*39*.*2*	*0*.*000*	*0*.*013*	*6*.*4*	*0*.*000*	*0*.*002*	*48*.*5*	*0*.*000*	*0*.*016*	2.9	0.034	0.001
Training experience	3	1.0	0.413	0.000	*117*.*3*	*0*.*000*	*0*.*037*	5.7	0.001	0.002	*6*.*2*	*0*.*000*	*0*.*002*	*141*.*9*	*0*.*000*	*0*.*063*
Owners’ gender	1	7.7	0.005	0.001	4.5	0.033	0.000	5.4	0.020	0.001	3.7	0.055	0.000	1.7	0.189	0.000
Owners’ age	3	*8*.*4*	*0*.*000*	*0*.*003*	0.4	0.747	0.000	*6*.*6*	*0*.*000*	*0*.*002*	1.9	0.122	0.001	5.7	0.001	0.003
Owners’ education	3	2.0	0.115	0.001	5.5	0.001	0.002	5.8	0.001	0.002	1.2	0.316	0.000	*19*.*0*	*0*.*000*	*0*.*009*
N of previous dogs	3	5.0	0.002	0.002	5.3	0.001	0.002	1.8	0.152	0.001	1.7	0.155	0.001	*30*.*2*	*0*.*000*	*0*.*014*
N of dogs in household	2	2.1	0.129	0.000	0.9	0.388	0.000	1.5	0.222	0.000	1.2	0.297	0.000	3.4	0.033	0.001
Where the dog is kept*	2	N/A												2.6	0.071	0.001
Hours spend walking*	2	N/A												*69*.*0*	*0*.*000*	*0*.*021*
Error		9144			9144			9144			9144			6351		

Non-significant main effects were not removed from the models. Significant effects at the level of p < 0.00037 are marked in italics. Factors marked with asterisks were investigated in Survey 2 only.

Aside from the dog group we found numerous associations between the demographic and dog keeping factors and the behaviour traits (for statistical details see Table 3), but there were no significant interactions between the dog group and any of the factors in any of the models.

Calmness: the 11 factors together accounted for 4.9% of the total variance in this trait. Five factors had significant associations after the Bonferroni correction: mixed-breeds were less calm than purebreds, older dogs were calmer, and neutered dogs were less calm. Dogs acquired before 12 weeks of age were calmer than dogs acquired at older age. Owners under the age of 18 years assessed their dogs as calmer than older owners’ assessments. From these factors, only dog group had a higher than 1% effect size (1.2%) (Table 3).

Trainability: the 11 factors together accounted for 10.3% of the total variance in this trait. Four factors had significant associations after the Bonferroni correction: mixed-breeds were more trainable than purebreds, older dogs were less trainable than younger ones, dogs acquired at > 1 year old were less trainable than dogs acquired at a younger age, and higher training level was associated with higher trainability assessment. From these four factors, three had a higher than 1% effect size: age (2.2%), age at acquisition (1.3%) and training level (3.7%) (Table 3).

Dog sociability: the 11 factors together accounted for 8.3% of the total variance in this trait. Four factors had significant associations after the Bonferroni correction: older dogs were less sociable towards other dogs than younger dogs; we found higher sociability in females than in males, and in dogs acquired between 2–12 weeks of age, than in dogs acquired at older ages. Owners younger than 30 years of age reported lower sociability in their dogs than older owners did. From these four factors, only age had a higher than 1% effect size (5.4%) (Table 3).

Boldness: the 11 factors together accounted for 4.1% of the total variance in this trait. Three factors had significant associations after the Bonferroni correction: males were reported to be bolder than females, dogs acquired between 2–12 weeks of age were bolder than dogs acquired at older ages, and dogs receiving only one training type were bolder than dogs receiving no training, or three or more types. From these three factors, two had a higher than 1% effect size: sex (1.4%) and age at acquisition (1.6%) (Table 3).

Problematic behaviour: the 13 factors together accounted for 15.4% of the total variance in this trait. Seven factors had significant associations after the Bonferroni correction: owners of purebreds, older dogs and females reported fewer behaviour problems in their dogs. Dogs with more training experiences displayed fewer behavioural problems (according to the owner). More educated and more experienced owners also reported that their dogs had fewer behaviour problems. Finally, owners who had longer walks with their dogs reported fewer behaviour problems. From these seven factors, four had a higher than 1% effect size: age (2.5%), training (6.3%), N of previous dogs (1.4%) and hours spend walking (2.1%) (Table 3).

## Discussion

In this study we demonstrated that purebred and mixed-breed dogs differ in some of their personality traits, and in the frequency of behaviour problems reported by the owner. Purebred and mixed-breed dogs are also kept differently within owners’ homes. In general, compared to how the owners of purebreds rated their dogs, mixed-breed dogs were perceived by their owners as less calm, and less sociable with other dogs, and their owners found their behaviour to be more problematic. However, the analyses of the effect sizes indicated small differences in all traits, with the possible exception of the calmness trait. Previous studies have reported similar behavioural differences between purebred and mixed-breed dogs, mainly in fearfulness, neuroticism and aggression-related behaviours [7,19,21,22].

We also found numerous differences between the purebred and mixed-breed dogs in their demographic and dog keeping characteristics: 12 from the 20 comparisons were significant after correcting for multiple comparisons. Mostly, demographic factors differed, e.g. more women keep mixed-breeds than men do, owners of mixed-breeds are less educated, are younger and have less experience with dogs. Probably related to the demographic differences, we also found differences in the dog keeping characteristics, e.g. mixed-breeds received less training, they were more likely to be kept only indoors, and as single dogs. However, we found no difference in the attitude and commitment of the owners (e.g. purpose of keeping, time spent with the dog, playing, giving gifts or allowing the dog on the bed), with the exception that mixed-breeds’ owners walked their dogs for longer than the owners of purebreds.

However, for the majority of the factors, the magnitude of the differences was too small to be relevant, regardless of statistical significance. Only two factors showed considerable (> 10%) differences between purebreds and mixed-breeds: the dogs’ neuter status and their age at acquisition. Mixed-breed dogs were more likely to be neutered, less likely to be acquired between 2–12 weeks of age, and more likely to be adopted at an older age than purebred dogs.

When we investigated the relationship between the demographic and environmental factors and the behaviour traits, we found that 9 out of the 12 factors were significantly associated with at least one behaviour trait. Most of these associations were in agreement with the results from our previous study by Kubinyi et al. [9]. We found no significant interactions between the dog group and any demographic or dog keeping factors in any of the behaviour traits, indicating that these factors have similar associations with the behaviour both in purebreds and in mixed-breeds.

However, it is worth considering that these three results, i.e. the dog group differences in behaviour, the dog group differences in demographic/dog keeping factors, and the associations between the behavioural traits and the demographic/dog keeping factors are not independent from each other. It is possible that we found behaviour differences between the dog groups only because mixed-breeds and purebreds show a different distribution regarding numerous demographic/dog keeping factors, which are in turn linked to the measured behaviour traits. To rule out this possibility, we used statistical models which controlled for all demographic and dog keeping factors where dog group differences were found.

Contrary to our earlier result, where mixed-breeds were found to have lower sociability towards other dogs, the General Linear Model indicated no significant effect of the dog group on the dog sociability trait. In this model, the strongest predictors of dog sociability were the dog’s age, sex, age at acquisition, and owners’ age: older dogs, dogs acquired at an older age, males and dogs of younger owners had lower dog sociability ratings. Since in our sample, mixed-breeds were older, were acquired at an older age and had younger owners than purebreds, the lower sociability of the mixed-breeds we found in the initial behaviour comparison, seems to be an indirect result of these demographic differences. After controlling for them, we found no difference in dog sociability between the dog groups.

The opposite seems to be the case with the trainability trait. We found that mixed-breeds are more trainable than purebreds, but only after controlling for the demographic and dog keeping differences. Trainability was most strongly associated with dog’s age, age at acquisition, and training level: older dogs, dogs acquired at older ages, and dogs with a lower training level had lower trainability ratings. In this case, the characteristics of our sample (i.e. mixed-breeds were older, were acquired at an older age and received less training than purebreds), seems to mask the behavioural difference, therefore we only found higher trainability in mixed-breeds when we controlled for these demographic and dog keeping differences. In the cases of the boldness, calmness and problematic behaviour traits, the GLM analyses corroborate the initial behaviour comparisons. For boldness, neither the initial behaviour comparison nor the GLM analysis indicated a significant difference between the dog groups. For the calmness and problematic behaviour traits, the dog group remained as a significant predictor in the models even after controlling for the measured demographic and dog keeping factors. This suggests that these behaviour differences (i.e. less calm and more problematic behaviour in mixed-breed dogs), cannot be attributed solely to the environmental differences–at least not to those investigated in the current study.

Alternative explanations for the observed dog group differences, involve possible genetic differences. For example, Schneider et al. [21] suggested that dog breeders generally focus on producing individuals with desirable behaviour, resulting in more favourable behaviour characteristics, and a lower frequency of behaviour problems in purebreds compared to mixed-breeds. Gácsi et al. [30] also raised an interesting hypothesis involving genetic influences, which posits that the present day mixed-breeds may originate from a population that has been under continuous selection for independent survival skills. It is also possible that developing dogs with independent survival skills may not be favourable for breeders, thus the two hypotheses are linked to each other. Although we could not directly test any of these hypotheses in the current study, our results lend support to both of them. Breeders may selectively breed dogs that make good human companions, which includes being calmer and showing fewer behaviour problems. In contrast, for independent survival, more assertive and more nervous/alert behaviour could be advantageous, for example to solve problems independently, avoid strangers, other dogs, or possibly dangerous objects (cars, trains). We have to note however, that both outcomes define mixed-breeds as having at least one mixed-breed parent, and do not include dogs that are a mix of purebreds. We do not know the proportions of such individuals in our sample, so we were not able to make this definition.

Developmental effects, like early socialization, rearing environment and past experiences can also provide an explanation for the observed behaviour differences. Previous studies have shown that the amount of appropriate socialization during early development plays a large role in whether or not the dog develops behavioural problems, including fearfulness and aggression (reviewed in [31]). For example, dogs raised in a home with children were scored higher in energy level, excitability, and distractibility; and dogs that had been able to play with other dogs scored during development scored lower on separation-related behavior [32]. Mixed-breeds are highly overrepresented in shelters (e.g. 80%, [33]) and among stray dogs relinquished to shelters (75.2%, [34]), partly because puppies from unintended litters relatively frequently end up in a shelter or on the street. However, unintended litters rarely occur between dogs from the same breed. Thus mixed-breeds, which were adopted at an older age likely originated from shelters or from the street, where they lived in a completely different environment than dogs raised by their owners [35,36]. The results of numerous studies provide strong evidence of a link between exposure to stressors associated with shelter life (like social and spatial restrictions), and the prevalence of undesirable behaviours in later life [23,37–39].

Our study is the first that was primarily aimed at addressing the possible behavioural and environmental differences between purebred and mixed-breed dogs, and as such, it is largely exploratory in nature. The most important limitation of the study is related to its subjectivity, as it was based on owner reports. Although the questionnaire method in general has proven to be a reliable and valid measurement, especially when complex behaviour traits are assessed (e.g. [40]), and our surveys showed good reliability, our study was still an indirect evaluation based on the owners’ memories of their dogs’ behaviour. Therefore, differences in the owners’ attitude towards purebreds and mixed-breeds may bias our results. Although it was not possible for us to determine whether the dogs were really purebred, or just that the owners believed them to be, some owners may value a dog's pedigree as a status symbol, or believe that a pedigree means that the dog possesses superior behavioural characteristics. Such owners may have a tendency to “overrate” their dogs, or report less problematic behaviours. However, we believe that these biases may be minimized by large sample sizes. Moreover, our owners represented a self-nominated, convenience sample, who are interested in their dogs’ behaviour, and all our subjects were living in families, so we have no information available about the shelter, stray or feral dog populations. Finally, no study could possibly measure all the relevant environmental and dog keeping factors in association with the dogs’ behaviour; for example, it would have been interesting to determine where the dogs in the present study were obtained, and how this may have affected their behavioural traits.

## Conclusions

We found numerous behavioural, demographic, and environmental differences between purebred and mixed-breed dogs based on the owners’ reports. However, many of these were small and their biological relevance is questionable. The dog sociability and trainability traits were more strongly influenced by the environmental characteristics of our sample: the mixed-breeds’ lower sociability towards other dogs we initially found, seemed to be an indirect result of the environmental differences between purebreds and mixed-breeds, while the mixed-breeds’ higher trainability seemed to be masked by these environmental differences. However the differences we found in calmness and problematic behaviour traits were less influenced by environmental factors, and were more likely due to early socialization and / or genetic effects. Therefore, our results suggest that care should be taken when using mixed-breeds as control dogs in breed or species comparison studies. Despite their diversity in morphological terms, mixed-breed dogs may not represent the ‘average’ dogs, but instead constitute a special group of dogs with characteristic behaviour traits.

## Supporting information

S1 Table The list of breeds included in the study. (PDF)

S2 Table Descriptive data of the purebred group in Survey 1 (S1) and Survey 2 (S2). (PDF)

S3 Table Questionnaire items in Survey 1 (see in [9]). (PDF)

S4 Table Questionnaire items in Survey 2. (PDF)

S5 Table Detailed information about the subjects and data. (XLSX)
