# Supplementary material for: Owner perceived differences between mixed-breed and purebred dogs
Source: PLoS One. 2017 Feb 21;12(2):e0172720. doi: 10.1371/journal.pone.0172720 (PMC5319786; doi:10.1371/journal.pone.0172720)
Supplement: S1 Table — (PDF) [file pone.0172720.s001.pdf]

S1 Table. The list of breeds included in the study: their sample sizes in Survey 1 (N in S1) and Survey 2 (N in S2), N of individuals in both surveys (N both Surveys), N of individuals in the merged population (S1+S2=N Total), sex ratio (ratio of males (%)), mean age (in years), and distribution according to FCI categorization (FCI group).

Breed	N in S1	N in S2	N both surveys	N Total	ratio of males (%)	mean age (in years)	FCI group
Affenpinscher	2	2	1	3	66.7%	6.0	2
Afghan Hound	3	2	0	5	40.0%	7.8	10
Airedale Terrier	31	37	1	67	61.2%	3.7	3
Akbash Dog	3	2	1	4	75.0%	2.7	NR
Akita	22	6	0	28	46.4%	3.8	5
Alaskan Malamute	11	8	1	18	77.8%	4.6	5
Alpine Dachsbracke	4	1	0	5	100%	5.7	6
Altdeutscher Hütehund	21	30	0	51	60.8%	3.4	NR
American Bulldog	60	15	0	75	61.3%	2.1	NR
American Cocker Spaniel	16	1	0	17	64.7%	5.7	8
American Staffordshire Terrier	60	26	2	84	58.3%	4.7	3
Andalusian Hound Maneto	0	1	0	1	100%	3.0	NR
Andalusian Podenco	2	2	0	4	75.0%	5.3	NR
Appenzell Cattle Dog	37	18	0	55	63.6%	2.7	2
Australian Cattle Dog	11	10	0	21	42.9%	1.9	1
Australian Kelpie	3	5	0	8	62.5%	2.1	1
Australian Shepherd	60	37	0	97	64.9%	2.5	1
Australian Silky Terrier	0	2	0	2	100%	2.0	3
Australian Terrier	5	10	1	14	42.9%	3.4	3
Austrian Black and Tan Hound	2	1	0	3	33.3%	2.1	6
Austrian Pinscher	2	4	0	6	16.7%	6.1	2
Azawakh	0	2	0	2	100%	1.7	10
Bandog	2	0	0	2	50.0%	10.5	NR
Basenji	4	3	0	7	85.7%	5.5	5
Basque Shepherd Dog	1	2	1	2	50.0%	3.0	NR
Basset Fauve de Bretagne	1	1	0	2	50.0%	5.0	6
Basset Hound	8	5	0	13	38.5%	4.0	6
Bavarian Mountain Scent Hound	14	7	0	21	71.4%	3.2	6
Beagle	60	37	0	97	56.7%	2.4	6
Bearded Collie	58	33	1	90	54.4%	4.4	1
Bedlington Terrier	1	1	0	2	50.0%	4.5	3
Belgian Shepherd Dog - Groenendael	9	9	1	17	47.1%	4.1	1
Belgian Shepherd Dog - Malinois	60	32	1	91	56.0%	3.1	1
Belgian Shepherd Dog - not specified	24	15	0	39	53.8%	3.7	1
Belgian Shepherd Dog - Tervuren	6	6	0	12	58.3%	4.8	1
Berger de Beauce	12	3	0	15	73.3%	2.9	1
Bernese Mountain Dog	60	37	0	97	56.7%	3.3	2
Bichon Frise	13	12	1	24	58.3%	3.2	9
Biewer Terrier	9	6	0	15	66.7%	1.6	NR
Blue Gascony Basset	1	2	0	3	100%	3.4	6
Boerboel	1	10	0	11	54.5%	2.5	NR

Breed	N in S1	N in S2	N both surveys	N Total	ratio of males (%)	mean age (in years)	FCI group
Bohemian Shepherd	1	0	0	1	100%	4.0	NR
Bolognese	8	6	1	13	76.9%	3.4	9
Bolonka Zwetna	40	16	0	56	60.7%	1.8	NR
Border Collie	60	37	0	97	57.7%	2.9	1
Border Terrier	14	21	2	33	54.5%	4.3	3
Borzoi	5	2	0	7	71.4%	4.1	10
Boston Terrier	5	8	0	13	76.9%	2.2	9
Bouvier des Flandres	8	10	2	16	56.3%	4.0	1
Boxer	60	37	0	97	52.6%	2.9	2
Brazilian Terrier	1	0	0	1	0%	2.0	3
Briard	39	37	0	76	60.5%	3.6	1
Brittany Spaniel	11	22	0	33	54.5%	4.0	7
Bull Terrier	20	15	0	35	57.1%	3.6	3
Bulldog	60	37	0	97	51.5%	2.3	2
Bullmastiff	8	19	0	27	59.3%	3.1	2
Cairn Terrier	31	28	3	56	48.2%	5.2	3
Canaan Dog	4	2	0	6	66.7%	2.0	5
Canarian Warren Hound	5	3	1	7	28.6%	5.2	5
Castro Laboreiro Dog	0	3	0	3	66.7%	2.6	2
Catahoula Cur	0	1	0	1	0%	0.8	NR
Catalan Sheepdog	8	1	0	9	33.3%	2.9	1
Caucasian Shepherd Dog	9	3	0	12	83.3%	2.1	2
Cavalier King Charles Spaniel	25	36	1	60	61.7%	2.5	9
Central Asian Shepherd Dog	2	1	0	3	33.3%	3.0	2
Cesky Terrier	0	2	0	2	50.0%	3.0	3
Chesapeake Bay Retriever	4	1	0	5	80.0%	4.4	8
Chihuahua	60	37	0	97	57.7%	2.5	9
Chinese Crested Dog	8	4	1	11	90.9%	3.6	9
Chow Chow	6	3	0	9	66.7%	6.0	5
Coarse-haired Styrian Hound	1	0	0	1	0%	4.0	6
Collie Rough	60	37	0	97	55.7%	4.2	1
Collie Smooth	3	10	0	13	61.5%	1.8	1
Continental Bulldog	4	3	1	6	83.3%	2.2	NR
Continental Toy Spaniel	7	11	0	18	44.4%	2.9	9
Coton de Tulear	23	16	1	38	55.3%	2.9	9
Cretan Hound	1	2	0	3	0%	2.7	NR
Croatian Shepherd Dog	2	2	0	4	75.0%	1.7	2
Czechoslovakian Wolfdog	3	6	1	8	50.0%	2.6	1
Dachshund - Long-haired	6	7	0	13	53.8%	3.1	4
Dachshund - Miniature	30	15	0	45	48.9%	2.4	4
Dachshund - Smooth-haired	60	37	0	97	61.9%	4.5	4
Dachshund - Wire-haired	60	37	0	97	64.9%	4.3	4
Dalmatian	60	37	0	97	61.9%	4.0	6
Dandie Dinmont Terrier	1	0	0	1	100%	1.0	3
Danish-Swedish Farmdog	0	1	0	1	100%	1.0	2

Breed	N in S1	N in S2	N both surveys	N Total	ratio of males (%)	mean age (in years)	FCI group
Deerhound	1	3	0	4	50.0%	4.7	10
Dobermann	60	37	0	97	64.9%	3.0	2
Dogo Argentino	10	9	0	19	47.4%	3.8	2
Dogo Canario	14	18	3	29	62.1%	2.2	2
Dogue de Bordeaux	24	12	0	36	36.1%	2.6	2
Drentsche Partridge Dog	0	1	0	1	0%	4.0	7
Dutch Schapendoes	11	4	1	14	64.3%	2.6	1
Dutch Shepherd Dog	13	9	0	22	45.5%	4.3	1
Elo	18	19	0	37	51.4%	2.5	NR
English Cocker Spaniel	60	37	0	97	57.7%	4.0	8
English Foxhound	0	1	0	1	100%	2.0	6
English Pointer	11	12	1	22	54.5%	3.5	7
English Setter	11	11	0	22	54.5%	5.1	7
English Springer Spaniel	5	9	1	13	69.2%	3.6	8
English Toy Terrier	1	2	0	3	33.3%	4.5	3
Entlebuch Cattle Dog	21	20	1	40	55.0%	3.6	2
Estrela Mountain Dog	0	1	0	1	0%	1.7	2
Eurasian	25	23	3	45	66.7%	4.3	5
Eurohound	2	0	0	2	50.0%	2.7	NR
Fila Brasileiro	3	5	0	8	62.5%	2.2	2
Finnish lapphund	1	0	0	1	0%	5.0	5
Flat Coated Retriever	21	37	1	57	49.1%	2.5	8
Fox Terrier - Smooth-haired	9	5	0	14	71.4%	4.5	3
Fox Terrier - Wire-haired	32	34	1	65	58.5%	4.3	3
French Bulldog	60	37	0	97	70.1%	1.9	9
French Pointing Dog - Pyrenean type	1	1	0	2	50.0%	4.0	7
Garafian Shepherd	0	2	0	2	100%	2.5	NR
German Beardog	2	2	0	4	75.0%	2.2	NR
German Hound	10	6	0	16	81.3%	2.7	6
German Hunting Terrier	13	11	0	24	66.7%	3.2	3
German Long-haired Pointing Dog	7	2	0	9	44.4%	3.5	7
German Pinscher	24	33	1	56	64.3%	3.1	2
German Shepherd Dog	60	37	0	97	52.6%	4.0	1
German Short-haired Pointing Dog	24	15	0	39	69.2%	3.6	7
German Spaniel	3	1	0	4	25.0%	3.0	8
German Spitz - Giant	0	3	0	3	66.7%	1.3	5
German Spitz - Medium	18	9	0	27	40.7%	4.0	5
German Spitz - Miniature	10	6	0	16	62.5%	3.1	5
German Spitz - Wolfspitz	14	7	0	21	71.4%	5.2	5
German Wire-haired Pointing Dog	26	14	0	40	55.0%	3.9	7
Giant Schnauzer	60	37	0	97	61.9%	3.7	2
Golden Retriever	60	37	0	97	56.7%	3.6	8
Gordon Setter	11	19	0	30	60.0%	3.4	7
Grand Basset Griffon Vendeen	1	5	0	6	50.0%	3.6	6
Great Anglo-French Tricolour Hound	0	1	0	1	100%	4.0	6

Breed	N in S1	N in S2	N both surveys	N Total	ratio of males (%)	mean age (in years)	FCI group
Great Dane	60	37	0	97	55.7%	2.9	2
Great Swiss Mountain Dog	22	22	3	41	61.0%	3.8	2
Greyhound	2	5	1	6	33.3%	5.7	10
Hanoverian Scenthound	6	1	1	6	83.3%	2.7	6
Harrier	1	0	0	1	100%	2.0	6
Havanese	60	37	3	94	53.2%	2.1	9
Heideterrier	1	0	0	1	0%	2.0	NR
Hellenic Hound	2	5	0	7	42.9%	2.6	6
Hovawart	60	37	0	97	60.8%	3.6	2
Hungarian Greyhund	3	2	1	4	75.0%	2.1	10
Transylvanian Scent Hound	0	1	0	1	100%	5.0	6
Hungarian Short-haired Pointer	60	37	0	97	54.6%	2.4	7
Hungarian Wire-haired Pointer	2	1	1	2	50.0%	5.5	7
Ibizan Podenco	6	5	0	11	63.6%	5.4	5
Icelandic Sheepdog	5	5	1	9	55.6%	5.0	5
Irish Red Setter	39	28	1	66	42.4%	4.2	7
Irish Soft Coated Wheaten Terrier	19	8	0	27	48.1%	4.5	3
Irish Terrier	17	12	0	29	51.7%	4.3	3
Irish Water Spaniel	1	0	0	1	0%	2.0	8
Irish Wolfhound	19	14	0	33	45.5%	2.9	10
Italian Corso Dog	11	28	1	38	60.5%	2.0	2
Italian Greyhound	1	0	0	1	100%	0.8	10
Italian Segugio	5	1	0	6	66.7%	3.8	6
Italian Spinone	0	1	0	1	0%	1.5	7
Italian Volpino	1	0	0	1	100%	1.0	5
Jack Russell Terrier	60	37	0	97	59.8%	2.3	3
Jämthund	1	0	0	1	100%	8.0	5
Japanese Chin	5	0	0	5	40.0%	4.5	9
Japanese Spitz	0	1	0	1	0%	2.0	5
Japanese Terrier	1	0	0	1	100%	0.3	3
Kangal	15	12	0	27	51.9%	3.3	2
Karelian Bear Dog	0	1	0	1	100%	4.0	5
Kerry Blue Terrier	2	1	0	3	33.3%	2.5	3
King Charles Spaniel	3	0	0	3	100%	10.0	9
Komondor	1	2	0	3	0%	1.3	1
Korean Jindo Dog	1	1	1	1	0%	2.0	5
Kromfohrländer	60	19	3	76	60.5%	3.2	9
Kuvasz	4	10	0	14	50.0%	4.2	1
Labrador Retriever	60	37	0	97	54.6%	2.5	8
Lakeland Terrier	5	3	0	8	25.0%	3.1	3
Landseer	16	14	1	29	51.7%	2.4	2
Large Münsterländer	7	5	0	12	75.0%	4.9	7
Leonberger	22	13	0	35	60.0%	2.9	2
Lhasa Apso	20	5	1	24	58.3%	5.7	9
Little Lion Dog	2	5	0	7	71.4%	4.4	9

Breed	N in S1	N in S2	N both surveys	N Total	ratio of males (%)	mean age (in years)	FCI group
Majorca Mastiff	2	0	0	2	50.0%	4.3	2
Majorca Ratter	0	2	0	2	100%	6.0	NR
Majorca Shepherd Dog	4	2	0	6	33.3%	4.3	1
Maltese	60	37	0	97	55.7%	3.8	9
Manchester Terrier	10	4	2	12	58.3%	4.8	3
Maremma Sheepdog	9	3	0	12	50.0%	3.0	1
Markiesje	1	0	0	1	100%	1.4	NR
Mastiff	3	13	3	16	50.0%	3.5	2
Medium Poodle	45	28	0	73	67.1%	4.9	9
Miniature Australian Shepherd	6	2	0	8	62.5%	1.5	1
Miniature Bull Terrier	11	20	2	29	62.1%	2.1	3
Miniature Pinscher	42	37	1	78	55.1%	2.0	2
Miniature Poodle	31	14	0	45	55.6%	4.1	9
Miniature Schnauzer	18	19	1	36	52.8%	4.3	2
Mudi	6	1	0	7	71.4%	4.1	1
Neapolitan Mastiff	2	6	0	8	62.5%	4.4	2
Nederlandse Kooikerhondje	9	8	0	17	64.7%	3.3	8
Newfoundland	42	23	2	63	54.0%	3.7	2
Norfolk Terrier	5	6	0	11	63.6%	3.8	3
Norwegian Buhund	0	1	0	1	0%	1.5	5
Norwegian Lundehund	0	1	0	1	0%	4.0	5
Norwich Terrier	1	5	0	6	50.0%	3.0	3
Nova Scotia Duck Tolling Retriever	6	12	1	17	70.6%	4.5	8
Old English Sheepdog	12	17	2	27	55.6%	4.3	1
Otterhound	2	1	0	3	33.3%	5.3	6
Parson Russell Terrier	60	37	0	97	57.7%	3.0	3
Patterdale Terrier	3	1	0	4	0%	3.5	NR
Pekingese	21	9	2	28	53.6%	3.5	9
Perro Bardino	2	4	0	6	50.0%	2.8	NR
Petit Basset Griffon Vendeen	2	2	0	4	50.0%	4.6	6
Picardy Sheepdog	4	6	1	9	88.9%	2.8	1
Pit Bull Terrier	58	21	1	78	59.0%	4.9	NR
Podenco - not specified	22	33	2	53	50.9%	3.8	5
Polish Greyhound	1	1	0	2	100%	4.8	10
Polish Hound	4	1	1	4	75.0%	1.2	6
Polish Lowland Sheepdog	23	16	0	39	51.3%	3.9	1
Portuguese Pointing Dog	3	1	0	4	50.0%	2.9	7
Portuguese Warren Hound	4	3	0	7	28.6%	2.4	5
Portuguese Water Dog	2	3	0	5	40.0%	1.6	8
Prague Ratter	8	12	0	20	40.0%	1.3	NR
Pudelpointer	2	1	0	3	66.7%	4.0	7
Pug	60	37	0	97	59.8%	2.2	9
Puli	2	4	0	6	33.3%	1.6	1
Pumi	1	1	0	2	100%	5.3	1
Pyrenean Mastiff	3	0	0	3	100%	3.1	2

Breed	N in S1	N in S2	N both surveys	N Total	ratio of males (%)	mean age (in years)	FCI group
Pyrenean Mountain Dog	5	3	1	7	42.9%	3.4	2
Pyrenean Sheepdog	27	5	0	32	65.6%	4.3	1
Ratonero - not specified	4	3	0	7	28.6%	2.1	NR
Ratonero Bodeguero Andaluz	0	2	0	2	0%	1.3	NR
Rhodesian Ridgeback	60	37	0	97	59.8%	2.2	6
Romagna Water Dog	4	4	0	8	50.0%	1.7	8
Romaner Antikdogge	3	1	0	4	50.0%	1.4	NR
Romanian Shepherd Dog	1	0	0	1	0%	0.3	2
Rottweiler	60	37	0	97	52.6%	3.8	2
Russian Black Terrier	8	7	2	13	61.5%	4.3	2
Russian Spaniel	1	0	0	1	0%	7.0	NR
Saarloos Wolfhond	4	1	0	5	80.0%	3.9	1
Saint-Usuge Spaniel	2	1	1	2	0%	4.3	NR
Saluki	4	6	0	10	60.0%	3.4	10
Samoyed	4	5	0	9	33.3%	4.2	5
Schipperke	1	1	0	2	50.0%	4.4	1
Schnauzer	18	14	1	31	61.3%	3.7	2
Scottish Terrier	10	6	1	15	33.3%	4.3	3
Sealyham Terrier	1	0	0	1	100%	3.0	3
Serbian Hound	0	1	0	1	0%	0.5	6
Shar Pei	24	13	0	37	64.9%	1.7	2
Shetland Sheepdog	33	19	0	52	53.8%	3.1	1
Shiba	46	11	2	55	41.8%	2.8	5
Shih Tzu	41	33	0	74	64.9%	3.0	9
Shiloh Shepherd Dog	1	0	0	1	0%	2.0	NR
Siberian Husky	60	37	0	97	53.6%	4.6	5
Sloughi	1	1	0	2	100%	2.8	10
Slovakian Chuvach	1	2	1	2	50.0%	7.0	1
Slovakian Hound	2	0	0	2	100%	5.8	6
Small Münsterländer	60	34	1	93	48.4%	4.5	7
Spanish Alano	2	2	0	4	50.0%	2.2	NR
Spanish Greyhound	30	26	1	55	61.8%	4.0	10
Spanish Mastiff	2	1	0	3	33.3%	6.9	2
Spanish Water Dog	6	9	0	15	53.3%	2.7	8
St. Bernard	22	6	0	28	71.4%	3.6	2
Staffordshire Bull Terrier	16	4	0	20	35.0%	5.8	3
Staffordshire Terrier - not specified	48	13	0	61	45.9%	4.5	3
Standard Poodle	8	7	1	14	50.0%	5.5	9
Swiss Hound	1	4	0	5	40.0%	3.8	6
Tatra Shepherd Dog	4	2	0	6	83.3%	1.5	1
Terrier - not specified	28	25	0	53	69.8%	4.9	3
Thai Ridgeback Dog	1	3	0	4	75.0%	2.6	5
Tibetan Spaniel	1	4	0	5	80.0%	2.7	9
Tibetan Terrier	58	37	0	95	55.8%	3.4	9
Tosa	3	1	0	4	50.0%	2.3	2

Breed	N in S1	N in S2	N both surveys	N Total	ratio of males (%)	mean age (in years)	FCI group
Toy Poodle	2	4	0	6	50.0%	1.6	9
Tyrolean Hound	1	1	0	2	50.0%	1.6	6
Wäller	6	0	0	6	33.3%	7.3	NR
Weimaraner	52	37	0	89	59.6%	2.7	7
Welsh Corgi Cardigan	3	0	0	3	33.3%	3.5	1
Welsh Corgi Pembroke	1	1	1	1	100%	7.0	1
Welsh Springer Spaniel	2	1	0	3	0%	1.0	8
Welsh Terrier	13	17	1	29	69.0%	2.9	3
West Highland White Terrier	60	37	0	97	59.8%	5.2	3
West Siberian Laika	1	2	0	3	66.7%	4.5	5
Whippet	29	32	2	59	62.7%	3.5	10
White Swiss Shepherd Dog	60	37	0	97	46.4%	2.9	2
Wire-haired Pointing Griffon Korthals	5	3	0	8	37.5%	4.3	7
Xoloitzcuintle	3	3	0	6	33.3%	1.7	5
Yorkshire Terrier	60	37	0	97	56.7%	3.7	3
Yugoslavian Shepherd Dog	0	1	0	1	0%	5.0	2
**Purebred Total**	**4593**	**3199**	**92**	**7700**	**57.2%**	**3.4**	**-**
**Mixed-breed**	**4593**	**3185**	**87**	**7691**	**52.7%**	**3.7**	**-**

To control for the popularity of the breeds, a maximum for the number of individuals per breed was established (N = 60 for S1, N = 37 for S2). For the final dataset, a random sample was selected from the breeds with more representatives than these cut-off points. When calculating the total number (N Total), individuals in both surveys were counted only once.

The numbers in the FCI group column represents:

1: Sheepdogs and cattle dogs (except Swiss cattle dogs); 2: Pinscher and schnauzer – molossoid, Swiss mountain and cattle dogs; 3: Terriers; 4: Dachshunds; 5: Spitz and primitive types; 6: Scent hounds and related breeds; 7: Pointing dogs; 8: Retrievers, flushing dogs, water dogs; 9: Companion and toy dogs; 10: Sighthounds; NR: not recognized by the FCI.
