# Supplementary material for: Owner perceived differences between mixed-breed and purebred dogs
Source: PLoS One. 2017 Feb 21;12(2):e0172720. doi: 10.1371/journal.pone.0172720 (PMC5319786; doi:10.1371/journal.pone.0172720)
Supplement: S2 Table — (PDF) [file pone.0172720.s002.pdf]

S2 Table. Descriptive data of the purebred group in Survey 1 (S1) and Survey 2 (S2).

Survey 1

Descriptive characteristics	N of breeds	% of breeds	N of dogs	% of dogs
**a) N of dogs / breed**				
N = 60 dogs (the established maximum)	37	14.6%	2220	48.3%
N = 50-59 dogs	4	1.6%	226	4.9%
N = 40-49 dogs	6	2.4%	304	6.6%
N = 30-39 dogs	10	3.9%	333	7.3%
N = 20-29 dogs	20	7.9%	560	12.2%
N = 10-19 dogs	35	13.8%	471	10.3%
N = 1-9 dogs	142	55.9%	479	10.4%
**b) FCI group**				
Sheepdogs and cattle dogs (except Swiss cattle dogs)	37	14.6%	654	14.2%
Pinscher and schnauzer – molossoid. Swiss mountain and cattle dogs	42	16.5%	979	21.3%
Terriers	30	11.8%	646	14.1%
Dachshunds	4	1.6%	156	3.4%
Spitz and primitive types	23	9.1%	275	6.0%
Scent hounds and related breeds	22	8.7%	248	5.4%
Pointing dogs	17	6.7%	332	7.2%
Retrievers, flushing dogs, water dogs	15	5.9%	259	5.6%
Companion and toy dogs	25	9.8%	686	14.9%
Sighthounds	12	4.7%	99	2.2%
Breeds not recognised by the FCI	27	10.6%	259	5.6%
**Purebred group total**	**254**		**4593**	

Survey 2

Descriptive characteristics	N of breeds	% of breeds	N of dogs	% of dogs
**a) N of dogs / breed**				
N = 37 dogs (the established maximum)	38	15.1%	1406	44.0%
N = 30-36 dogs	9	3.6%	330	10.3%
N = 20-29 dogs	14	5.6%	361	11.3%
N = 10-19 dogs	42	16.7%	617	19.3%
N = 1-9 dogs	148	59.0%	485	15.2%
**b) FCI group**				
Sheepdogs and cattle dogs (except Swiss cattle dogs)	36	14.3%	433	13.5%
Pinscher and schnauzer - molossoid and Swiss mountain and cattle dogs	43	17.1%	717	22.4%
Terriers	28	11.2%	466	14.6%
Dachshunds	4	1.6%	96	3.0%
Spitz and primitive types	25	10.0%	185	5.8%
Scent hounds and related breeds	23	9.2%	159	5.0%
Pointing dogs	19	7.6%	245	7.7%
Retrievers, flushing dogs, water dogs	14	5.6%	197	6.2%
Companion and toy dogs	23	9.2%	443	13.8%
Sighthounds	12	4.8%	96	3.0%
Breeds not recognised by the FCI	24	9.6%	162	5.1%
**Purebred group total**	**251**		**3199**	

To control for the popularity of the breeds, a maximum for the number of individuals per breed was established (N = 60 for S1, N = 37 for S2). For the final dataset, a random sample was

selected from the breeds with more representatives than these cut-off points. The table presents two characteristics of the purebred group: a) the numbers of breeds within different frequency categories, and b) the distribution of the breeds and individuals among the FCI breed groups.
