# Supplementary material for: Owner perceived differences between mixed-breed and purebred dogs
Source: PLoS One. 2017 Feb 21;12(2):e0172720. doi: 10.1371/journal.pone.0172720 (PMC5319786; doi:10.1371/journal.pone.0172720)
Supplement: S3 Table — (PDF) [file pone.0172720.s003.pdf]

S3 Table. Questionnaire items in Survey 1 (see in Kubinyi et al., 2009).

**Calmness**	**Trainability**
Is calm, even in ambiguous situations	Is ingenious, inventive when seeks hidden food or toy
Can be stressed easily*	Is intelligent, learns quickly
Is emotionally balanced, not easy to rile	Is very easy to warm up to a new toy
Is cool-headed even in stressful situations	Is not much interested except in eating and sleeping*
Is sometimes anxious and uncertain*	Often does not understand what was expected from him/her during playing*
Cronbach's alpha: 0.849	Cronbach's alpha: 0.711

**Dog sociability**	**Boldness**
Fights with conspecifics frequently*	Is rather cool, reserved*
Is ready to share toys with conspecifics	Is unassertive, aloof when unfamiliar persons enter the home*
Is bullying with conspecifics*	Is sometimes fearful, awkward*
Gets on well with conspecifics	
Cronbach's alpha: 0.738	Cronbach's alpha: 0.642

All items were scored using a 3-point scale

* reverse coded
