# Supplementary material for: Owner perceived differences between mixed-breed and purebred dogs
Source: PLoS One. 2017 Feb 21;12(2):e0172720. doi: 10.1371/journal.pone.0172720 (PMC5319786; doi:10.1371/journal.pone.0172720)
Supplement: S4 Table — (PDF) [file pone.0172720.s004.pdf]

S4 Table. Questionnaire items in Survey 2.

**Behaviour problems**
When my dog is let off leash, (s)he moves independent but regularly seeks eye contact with me and stays within sight.*
My dog is frequently stubborn and does not pay any attention to me. I let it go, as there is no use to anger myself.
We easily passed our BH§ examination.*
My dog knows his/her restrictions in the house, however has his/her own kennel, where (s)he can do what (s)he wants.*
My dog frequently pulls on the leash. I have tried everything but cannot control him/her.
When I arrive home my dog jumps up at me. I do not like it but (s)he does not give up.
When a visitor rings the bell my dog knows that (s)he is to stay back from the door.*
When somebody offers food to my dog, (s)he looks at me as if to ask whether (s)he is allowed to take it.*
It would be great if my dog were more obedient. However, I do not often feel the need to reprimand him/her.
When I arrive home my dog usually lays on the sofa. I cannot do anything about it.
There is no dominance problem in our family. My dog accepts every family member as a higher-ranking individual.*
My dog usually does not listen to me when I call him/her back. I get really angry about it.
Cronbach's alpha: 0.720

All items were scored using a 3-point scale

* reverse coded

§ BH is the abbreviation for Begleithundprüfung, i.e. "traffic-sure" companion dog test
